# ATRX large deletions and truncating mutations are associated with primary induction chemotherapy resistance in high-risk neuroblastoma: a multicenter propensity-weighted cohort study

**DOI:** 10.3389/fonc.2026.1857869

**Published:** 2026-07-22

**Authors:** Jing Sun, Bo Qian, Lihui Lu, Jing Zhou

**Affiliations:** 1 Department of Pediatrics, The Affiliated Taizhou People’s Hospital of Nanjing Medical University, Taizhou School of Clinical Medicine, Nanjing Medical University, Taizhou, China; 2 Department of Cardiothoracic Surgery, Children’s Hospital of Nanjing Medical University, Nanjing, China; 3 Department of Pediatrics, The Affiliated Hospital of Xuzhou Medical University, Xu Zhou, China; 4 Department of Oncology, The Affiliated Taizhou People’s Hospital of Nanjing Medical University, Taizhou School of Clinical Medicine, Nanjing Medical University, Taizhou, China

**Keywords:** ATRX, drug resistance, induction chemotherapy, neuroblastoma, structural variants

## Abstract

**Background:**

ATRX alterations are adverse prognostic factors in high-risk neuroblastoma patients. However, the precise impact of distinct ATRX mutation subtypes—particularly multiexon deletions versus missense mutations—on response to standard-of-care multiagent induction chemotherapy remains poorly defined.

**Methods:**

This multicenter retrospective cohort study evaluated newly diagnosed patients with high-risk neuroblastoma who underwent comprehensive molecular profiling and received platinum-containing multiagent induction chemotherapy between 2017 and 2022.center Following 1:2 subtype-based stratified random sampling, patients were categorized into the ATRX wild-type (WT), large deletion/truncating (LDT), and missense mutation subgroups. Multiple imputation by chained equations (MICE) and inverse probability of treatment weighting (IPTW) were applied to control for baseline confounders. The primary endpoint was the objective response rate (ORR), which was assessed by a blinded independent central review. Secondary endpoints included event-free survival (EFS), overall survival (OS), and cumulative incidence of progression (CIP).

**Results:**

The final weighted pseudocohort comprised 138 patients (WT, n = 92; LDT, n = 33; missense, n = 13). Compared with the WT group (71.0%), the LDT group exhibited a substantially lower ORR (36.0%; P < 0.001), with a primary induction-refractory rate of 64.0%. Patients with missense mutations demonstrated an intermediate ORR (60.0%, P = 0.285 vs. WT). At a median follow-up of 42.5 months, the LDT group had a lower 3-year EFS rate than the WT group (18.5% vs. 52.4%; P < 0.001) and a higher 3-year CIP (72.6% vs. 35.2%; subdistribution hazard ratio, 2.88; P < 0.001). The association between the LDT subtype and poor induction response persisted across clinical strata and was independent of 11q deletion or MYCN amplification status.

**Conclusions:**

Large ATRX deletions and truncating mutations identify an ultrahigh-risk molecular subtype of neuroblastoma characterized by primary resistance to conventional induction chemotherapy and accelerated progression. Detailed ATRX structural-variant subtyping may facilitate the early identification of patients at high risk of primary induction failure and supports the prospective evaluation of alternative or molecularly targeted induction strategies.

## 1 Introduction

Neuroblastoma is the most prevalent extracranial solid tumor in pediatric populations worldwide, representing approximately 8–10% of childhood malignancies and contributing to approximately 15% of pediatric cancer-related fatalities (1). Despite intensive multimodal therapy, the disease is characterized by remarkable clinical heterogeneity, with outcomes ranging from spontaneous regression in infants with favorable biology to relentless disease progression. Approximately half of neuroblastoma cases are classified as high risk according to the International Neuroblastoma Risk Group (INRG) classification system, which integrates clinical, histopathological, and molecular parameters for pretreatment risk stratification (2). Despite significant advances in multimodal therapeutic approaches, such as induction chemotherapy, surgical resection, myeloablative consolidation with autologous stem cell transplantation, radiotherapy, and maintenance immunotherapy with anti-GD2 monoclonal antibodies, long-term survival for patients with high-risk neuroblastoma remains suboptimal, with contemporary studies reporting a 5-year survival rate of approximately 50% (3).

Platinum-based compounds, including cisplatin and carboplatin, constitute essential components of all contemporary induction regimens for high-risk neuroblastoma, with alkylator and platinum doublets serving as the therapeutic backbone across multiple international cooperative group protocols (4). A recent systematic review of induction regimens published between 1995 and 2024 reported a median study-level end-induction response rate of 84.4%, yet the response rates have not improved substantially over the past three decades despite iterative refinements in dose intensity and schedule (4). Furthermore, the end-induction response has been established as a powerful independent predictor of the ultimate outcome, with patients who fail to achieve an objective response to frontline therapy facing a substantially elevated risk of subsequent relapse and death (5). While several recurrent genomic alterations, including MYCN amplification and 11q deletion, have been strongly associated with adverse outcomes, the molecular determinants governing differential responses to multiagent induction chemotherapy remain incompletely characterized (2).

Chromatin remodeling protein alpha-thalassemia/mental retardation syndrome X-linked (ATRX) has emerged as a recurrently altered gene in high-risk neuroblastoma, with mutations occurring in a recognizable patient subgroup characterized by older age at diagnosis, mutual exclusivity with MYCN amplification, and a chemotherapy-resistant, slowly progressive disease course (6, 7). Critically, recent studies have demonstrated that the ATRX mutational spectrum in neuroblastoma is uniquely enriched for multiexon deletions, with approximately 70% of ATRX aberrations comprising such structural variants, and an estimated 75% of these deletions are predicted to be in-frame alterations that produce internally truncated protein products (8, 9). This distinctive mutational pattern, which appears nearly exclusive to neuroblastoma compared with other pediatric malignancies, suggests potential functional divergence between mutation subtypes (9). Preliminary evidence indicates that patients harboring ATRX missense mutations may exhibit slightly better overall survival than those with other ATRX mutation types and that 11q deletions cooccur more frequently with multiexon deletions than with other ATRX mutation types or wild-type tumors do (8, 9). Moreover, ATRX aberrations are strongly correlated with alternative lengthening of the telomere pathway, and both ATRX aberrations and TERT rearrangements are enriched in 11q-deleted high-risk tumors (10).

Despite these advances in delineating the ATRX mutational landscape, a fundamental gap persists in understanding whether structurally distinct ATRX mutations are differentially associated with response to standard-of-care multiagent induction chemotherapy. Prior studies have largely aggregated all ATRX alterations into a single analytic category, potentially obscuring subtype-specific therapeutic vulnerabilities that could inform risk-adapted treatment strategies. Therefore, this multicenter retrospective cohort study was designed to evaluate whether ATRX mutation subtypes, defined according to the structural consequences of the underlying genomic alterations, were associated with differential objective response rates to multiagent induction chemotherapy, event-free survival, overall survival, and cumulative incidence of progression in children and adolescents with high-risk neuroblastoma. We hypothesized that, compared with missense mutations and wild-type ATRX status, large deletions and truncating mutations, particularly multiexon in-frame deletions, would be associated with primary resistance to conventional induction chemotherapy and poor clinical outcomes.

## 2 Methods

### 2.1 Study design and patient population

This multicenter retrospective cohort study was conducted using data extracted from the electronic medical records (EMR) and molecular genetics databases of The Affiliated Taizhou People’s Hospital of Nanjing Medical University (Taizhou, China) and Children’s Hospital of Nanjing Medical University (Nanjing, China) between January 2017 and December 2022. To minimize selection bias, comprehensive molecular profiling, including next-generation sequencing (NGS) and chromosomal microarray analysis (CMA) encompassing the ATRX gene, was implemented as a routine baseline screening protocol for all newly diagnosed high-risk neuroblastoma patients across participating centers starting in 2017. Given the relative rarity of ATRX mutations in high-risk neuroblastoma, a subtype-based stratified random sampling strategy was employed to guarantee adequate statistical power across mutation subgroups while maintaining the clinical feasibility of a blinded independent central review (BICR) for imaging. Specifically, all eligible patients harboring pathogenic ATRX mutations were exhaustively identified from the institutional databases; subsequently, a concurrent cohort of ATRX wild-type (WT) patients was established by randomly selecting cases at a 1:2 ratio (mutant to WT) from the available WT pool using computer-generated random numbers. The study protocol was reviewed and approved by the Institutional Review Board of The Affiliated Taizhou People’s Hospital of Nanjing Medical University (IRB Approval No: KY-2023-045-01) and the respective ethics committees of all the participating centers. A waiver of informed consent was granted by the Institutional Review Board of The Affiliated Taizhou People’s Hospital of Nanjing Medical University because of the retrospective, noninterventional nature of the data analysis, and all procedures were conducted in strict accordance with the Declaration of Helsinki and local data privacy regulations.

### 2.2 Study population

The study cohort was identified through a systematic query of the EMR systems and molecular genetics databases of the participating institutions using structured data filters, including relevant diagnostic codes for neuroblastoma and specific molecular testing logs. All potentially eligible patients diagnosed between the prespecified study dates were initially screened. Potential participants underwent a rigorous three-stage validation process: automated identification by clinical data warehouses on the basis of initial age and diagnostic criteria; manual review of medical, imaging, and genetic records by two independent pediatric oncologists using standardized electronic case report forms; and final adjudication by a senior pediatric oncologist in cases of diagnostic, molecular, or eligibility uncertainty. Any discrepancies encountered during the screening process were resolved through consensus discussion.

The inclusion criteria included the following: (1) a definitive diagnosis of neuroblastoma confirmed by histopathology or bone marrow cytology (11); (2) a classification as “high-risk” disease according to the International Neuroblastoma Risk Group (INRG) staging system (12); (3) age strictly between 18 months and 18 years at the time of diagnosis (13), encompassing both pediatric and adolescent populations; (4) initiation of a protocol-based multiagent induction chemotherapy regimen, generally planned for 6–8 cycles and including cisplatin or carboplatin as one component, with either an evaluable end-of-induction assessment or a documented clinical event before completion of induction (14); (5) the availability of complete NGS and CMA or fluorescence *in situ* hybridization (FISH) data clearly defining the ATRX mutation status and critical segmental chromosomal aberrations (specifically 11q deletion), with the strict prerequisite that all molecular data were derived from primary biopsy or surgical resection specimens obtained at initial diagnosis (prechemotherapy) to rigorously exclude acquired mutations induced by therapeutic selection pressure; and (6) the availability of complete clinical baseline data and comprehensive long-term follow-up records encompassing either the first systemic imaging evaluation following induction therapy or documentation of an early clinical endpoint (e.g., disease progression or toxicity-related death). The exclusion criteria included any of the following: (1) receipt of a nonprotocol or unclassifiable induction regimen, or insufficient treatment records to reconstruct the administered regimen and treatment exposure; (2) missing genetic sequencing data or sequencing depth insufficient for accurate mutation profiling and subtyping; (3) loss to follow-up prior to the first postinduction disease evaluation without any documented clinical endpoint; and (4) concurrent primary malignant neoplasms, advanced severe congenital anomalies, or other life-limiting conditions expected to confound treatment efficacy and survival assessments.

### 2.3 Induction chemotherapy

During the study period, patients received protocol-based multiagent induction chemotherapy according to the high-risk neuroblastoma treatment protocols adopted at the participating centers. The induction backbone generally consisted of alternating cyclophosphamide, doxorubicin, and vincristine (CAV) cycles and platinum–etoposide cycles, administered for 6–8 cycles before the end-of-induction response assessment. Cisplatin was the principal platinum agent, whereas carboplatin was used in selected patients according to the applicable institutional protocol or when cisplatin substitution was clinically required. Drug doses were calculated according to body surface area and were adjusted for body weight, organ function, and treatment-related toxicity in accordance with the relevant protocol.

For each patient, the induction regimen, platinum agent, number of completed cycles, cumulative platinum dose, dose reductions, treatment delays, and reasons for premature discontinuation were extracted from the medical records. Because cisplatin or carboplatin was administered as part of a multiagent regimen rather than as monotherapy, treatment response was evaluated as response to the overall induction chemotherapy regimen and was not attributed specifically to the platinum component.

### 2.4 Grouping criteria and variable definitions

Patients were stratified into three distinct molecular subgroups on the basis of the structural consequences of the identified ATRX alterations derived from next-generation sequencing breakpoint analysis, with variant pathogenicity rigorously evaluated in accordance with the American College of Medical Genetics and Genomics (ACMG) guidelines (15). The WT group comprised patients with no detectable pathogenic or likely pathogenic ATRX variants. The large deletion/truncated protein group included patients harboring massive structural variations, predominantly multiexon in-frame deletions (IFDs), as well as nonsense and frameshift mutations. In neuroblastoma, these characteristic multiexon IFDs frequently escape nonsense-mediated decay and are translated into abnormal, partially active truncated proteins rather than resulting in a complete loss of protein expression (16). The missense mutation group consisted of patients with pathogenic single-nucleotide variants that specifically disrupt critical functional regions, notably the ATRX-DNMT3-DNMT3L (ADD) domain or the helicase domain, thereby selectively impairing chromatin remodeling capabilities (17).

Baseline demographic, clinical, and tumor-specific variables, including age at diagnosis, sex, primary tumor location, baseline serum lactate dehydrogenase (LDH) levels, histological categorization according to the International Neuroblastoma Pathology Classification (INPC) (11), and baseline functional status assessed via the Lansky play-performance scale (18), were extracted from the electronic medical records. Key prognostic molecular and staging variables incorporated into the analysis included MYCN amplification status, 11q deletion status, and International Neuroblastoma Risk Group (INRG) stage (12). To rigorously control for the heterogeneity of pharmacokinetic exposure and chemotherapy adherence inherent in multicenter real-world data, the induction phase “dose exposure and compliance” was introduced as a core categorical adjustment variable. This metric was strictly dichotomized into “adequate exposure,” defined as the successful administration of at least 80% of the planned cumulative platinum dose with a total treatment delay of 4 weeks or less secondary to adverse events, and “suboptimal exposure,” defined as the receipt of less than 80% of the intended dose or a cumulative treatment delay exceeding 4 weeks due to severe myelosuppression or organ toxicity. Furthermore, the actual median cumulative platinum dose, calculated in milligrams per square meter of body surface area (mg/m²), was extracted as a continuous variable to verify dose equivalence across subgroups.

### 2.5 Study endpoints and imaging assessment criteria

The primary efficacy endpoint was the objective response rate (ORR), defined as the proportion of patients achieving a complete response (CR) or partial response (PR) at the end-of-induction assessment. Response assessments were performed according to the revised International Neuroblastoma Response Criteria (INRC) (19). Imaging studies were independently reviewed by two senior pediatric radiologists who were blinded to ATRX status and clinical outcomes. Bone marrow and other nonimaging disease assessments were reviewed using the corresponding pathology and clinical records. Disagreements were resolved by consensus or adjudication by a third reviewer.

Secondary endpoints included the primary induction-refractory rate, event-free survival (EFS), cumulative incidence of progression (CIP), and overall survival (OS). Primary induction-refractory disease was defined as MR, SD, or PD at the end-of-induction assessment, together with documented disease progression necessitating premature discontinuation or modification of the planned induction regimen. EFS was calculated from initiation of induction chemotherapy to the first occurrence of disease progression, relapse, a second malignant neoplasm, or death from any cause. OS was calculated from initiation of induction chemotherapy to death from any cause. For the CIP analysis, death without documented disease progression was treated as a competing event.

### 2.6 Sample size justification

Given the inherent rarity of large ATRX deletions and pathogenic variants in the target population, this study consolidated all pathogenic ATRX alterations into a pan-ATRX mutant (Mut) group, with exploratory subgroup analyses predefined on the basis of the predicted functional impact of the specific mutations. The sample size estimation was prospectively based on the primary efficacy endpoint of the objective response rate (ORR). On the basis of the outcomes reported in the HR-NBL1.5 trial, the anticipated ORR for high-risk neuroblastoma patients receiving standard platinum-based induction therapy was 70%, which served as the baseline expectation for the WT control group (14). Assuming an overall ATRX mutation frequency of 10% to 15%, a query of the participating multicenter databases was projected to yield approximately 45 eligible patients with ATRX mutations. Utilizing the predefined 1:2 subtype-based stratified random sampling strategy, approximately 90 WT patients were subsequently selected, resulting in a targeted total analytic cohort of 135 patients. Statistical power analyses indicated that detecting a clinically significant reduction in the ORR from 70% in the WT group to an anticipated 45% in the Mut group (corresponding to an approximate odds ratio of 0.35) with a two-sided alpha level of 0.05 would yield a statistical power of approximately 78%. Although this power was marginally below the conventional 80% threshold—a recognized constraint inherent to rare molecular subgroup analyses—the study was deemed adequately powered for initial real-world evaluation. To mitigate potential type II errors, statistical interpretation prioritized the estimation of effect sizes and their corresponding 95% confidence intervals over an exclusive reliance on null-hypothesis significance testing.

### 2.7 Statistical analysis

All the statistical analyses were executed using R software (version 4.5.1, R Foundation for Statistical Computing), with a two-sided P value of less than 0.05 predefined as the threshold for statistical significance. To address baseline covariate missingness (<20%), multiple imputation using chained equations (MICE) was performed, generating five imputed datasets that were subsequently pooled for downstream weighting and analyses in strict accordance with Rubin’s rules. To account for baseline imbalances, an inverse probability of treatment weighting (IPTW) approach was implemented using generalized propensity scores derived from multinomial logistic regression. The weighting model incorporated critical baseline confounders, specifically age at diagnosis, MYCN amplification status, 11q deletion status, and the previously defined chemotherapy dose exposure and compliance categorical metric. Covariate balance across the three molecular subgroups was rigorously verified by calculating the standardized mean difference (SMD), with an SMD of less than 0.1 considered indicative of optimal equilibrium. Concurrently, the variance inflation factor (VIF) was evaluated both before and after IPTW adjustment to assess multicollinearity. Differences in the ORR and primary induction-refractory rates across the weighted cohorts were assessed using weighted chi-square tests. Survival outcomes, including EFS and OS, were estimated using the weighted Kaplan–Meier method and statistically compared utilizing weighted log-rank tests. To accurately assess the absolute risk of disease progression while accounting for competing events, the Fine–Gray competing risk regression model was employed to calculate and compare the CIP, wherein non-tumor-specific mortality (e.g., severe chemotherapy-related toxicity) was strictly treated as a competing risk event. To establish the independent prognostic value of the ATRX mutation subtypes, a multivariable Cox proportional hazards regression model was constructed within the original (unweighted) cohort, simultaneously adjusting for ATRX genotype, MYCN amplification, 11q deletion, age, and chemotherapy exposure. This model included an interaction term between ATRX and 11q deletion status to assess the independence of the prognostic effects of ATRX. The proportional hazards (PH) assumption for the multivariable Cox models was rigorously verified using Schoenfeld residuals and graphical diagnostics. Furthermore, to overcome the statistical instability (quasicomplete separation) caused by the extreme mutual exclusivity between ATRX mutations and MYCN amplification, an independent, focused subgroup analysis was conducted exclusively within the MYCN-nonamplified (MYCN-NA) pure subset. Within this refined cohort, multivariable logistic regression was utilized to additionally adjust for 11q deletion status, thereby isolating the pure biological effect of ATRX alterations from this highly collinear genomic event. Finally, forest plots were generated to visualize the consistency of the resistance phenotype across various clinical strata, particularly when the truncating and IFD subgroups were compared. The robustness of the core findings was validated through prespecified sensitivity analyses: (1) weight trimming, which involved the truncation of extreme propensity score weights at the 1st and 99th percentiles; and (2) a perfect compliance cohort analysis, which restricted the evaluation exclusively to patients who achieved adequate exposure, thereby reducing potential confounding from suboptimal chemotherapy delivery in the response assessment.

## 3 Results

### 3.1 Patient screening and baseline clinical characteristics

**Figure 1:**
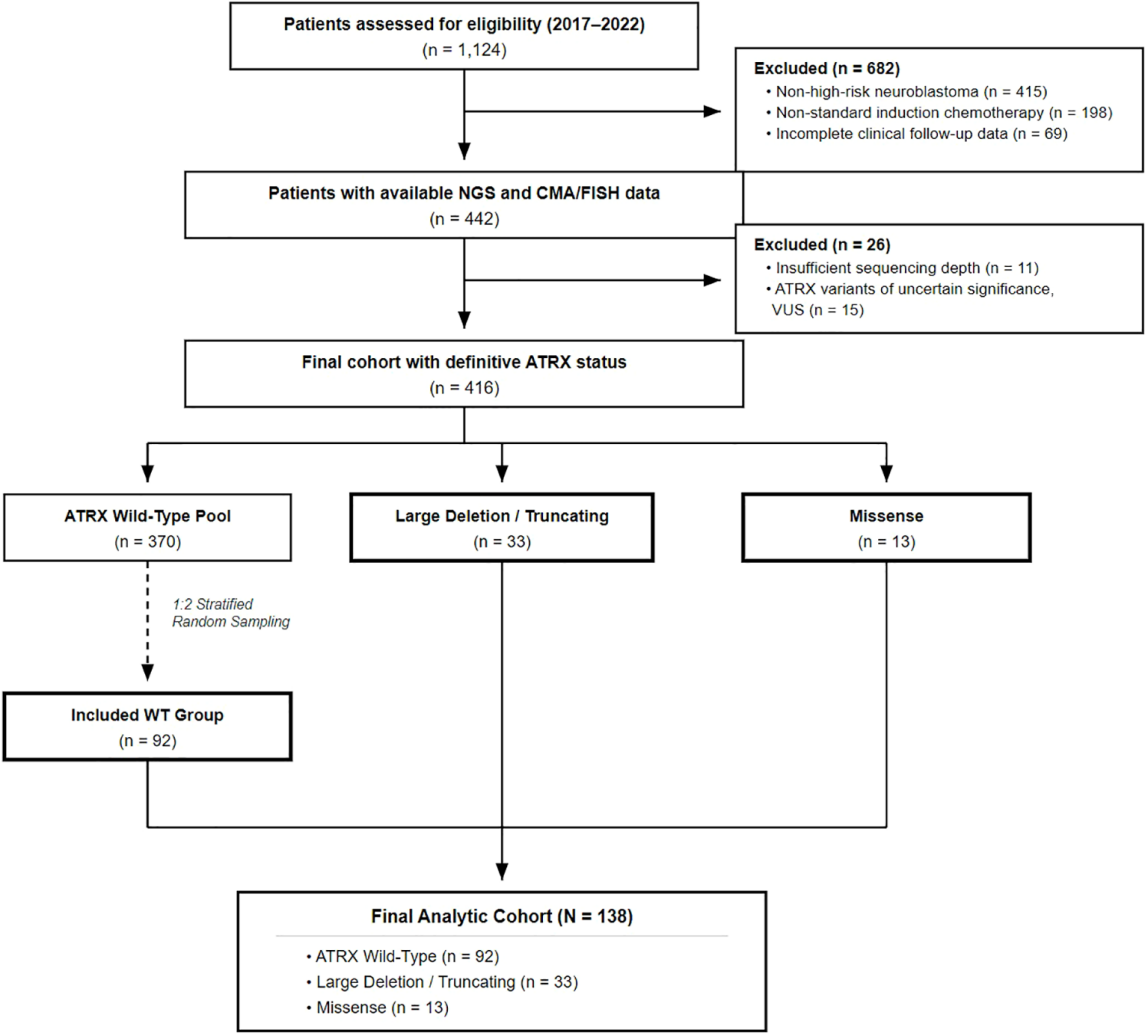
Flow diagram of subtype-based stratified random sampling, data imputation, and weighting.

A total of 138 eligible patients with high-risk neuroblastoma were included in the final analytic cohort, comprising 92 ATRX WT, 33 large deletion/truncating (inclusive of in-frame deletions), and 13 missense cases, successfully established via the predefined 1:2 subtype-based stratified random sampling strategy (**Figure 1**). In the original unweighted cohort, pronounced biological and demographic heterogeneity was observed among the three subgroups. Compared with the WT group, both mutant subgroups presented significantly older median ages at diagnosis (WT: 34.5; large deletion/truncating: 78.0; missense: 72.0 months; p < 0.001), substantially higher 11q deletion frequencies (23.9% vs. 72.7% and 53.8%; p < 0.001), and a near-complete absence of MYCN amplification (45.7% vs. 0.0% and 7.7%; p < 0.001). Following multiple imputation and IPTW, optimal equilibrium was achieved across all evaluated baseline covariates, including the Lansky Play Performance Scale, INPC categorization, and categorical dose exposure compliance, with all maximum standardized mean differences (Max SMDs) < 0.100 and VIFs consistently within safe thresholds (**Table 1**). Furthermore, the nonparametric comparison of cumulative platinum dose showed no statistically significant difference in delivered platinum exposure across the weighted WT, large deletion/truncating, and missense groups (395.0, 401.0, and 396.0 mg/m², respectively; p = 0.768), supporting the comparability of measured treatment exposure across the three groups.

**Table 1:** Comparison of baseline clinical and treatment characteristics among the three molecular subgroups before and after inverse probability of treatment weighting (IPTW).

Characteristic	Unweighted Cohort					Weighted Cohort (Post-IPTW)					
	WT (n = 92)	Large Deletion/Truncating (n = 33)	Missense (n = 13)	Statistic	p value	WT	Large Deletion/Truncating	Missense	Max SMD	Statistic	p value
Age at diagnosis (months), median (IQR)	34.5 (22.0-48.5)	78.0 (62.0-98.0)	72.0 (58.0-95.0)	H = 41.52	< 0.001	48.0 (32.0-68.0)	48.5 (33.5-67.0)	49.0 (33.0-66.0)	0.025	H = 0.12	0.941
Sex, n (%)											
Male	52 (56.5)	20 (60.6)	8 (61.5)	χ² = 0.28	0.869	58.20%	57.80%	58.00%	0.012	χ² = 0.01	0.995
Female	40 (43.5)	13 (39.4)	5 (38.5)			41.80%	42.20%	42.00%			
Primary tumor location, n (%)											
Adrenal	63 (68.5)	22 (66.7)	8 (61.5)	χ² = 0.32	0.852	66.80%	67.20%	67.00%	0.014	χ² = 0.01	0.995
Extra-adrenal	29 (31.5)	11 (33.3)	5 (38.5)			33.20%	32.80%	33.00%			
Baseline LDH (U/L), median (IQR)	850 (510-1280)	795 (485-1160)	810 (475-1180)	H = 0.85	0.653	825 (490-1210)	828 (500-1230)	830 (505-1240)	0.015	H = 0.09	0.955
INPC categorization, n (%)											
Favorable	15 (16.3)	4 (12.1)	2 (15.4)	χ² = 0.36	0.835	15.40%	15.80%	15.50%	0.011	χ² = 0.01	0.995
Unfavorable	77 (83.7)	29 (87.9)	11 (84.6)			84.60%	84.20%	84.50%			
Lansky play-performance scale, median (IQR)	80 (70-90)	80 (70-90)	80 (70-90)	H = 0.15	0.927	80 (70-90)	80 (70-90)	80 (70-90)	0	H = 0.00	1
MYCN amplification, n (%)											
Yes	42 (45.7)	0 (0.0)	1 (7.7)	χ² = 25.85	< 0.001	30.50%	30.20%	30.80%	0.015	χ² = 0.01	0.995
No	50 (54.3)	33 (100.0)	12 (92.3)			69.50%	69.80%	69.20%			
11q deletion, n (%)											
Yes	22 (23.9)	24 (72.7)	7 (53.8)	χ² = 24.12	< 0.001	38.60%	39.20%	38.90%	0.014	χ² = 0.01	0.995
No	70 (76.1)	9 (27.3)	6 (46.2)			61.40%	60.80%	61.10%			
INRG stage, n (%)											
L2	6 (6.5)	3 (9.1)	1 (7.7)	χ² = 0.31	0.856	7.30%	7.50%	7.40%	0.009	χ² = 0.00	1
M	86 (93.5)	30 (90.9)	12 (92.3)			92.70%	92.50%	92.60%			
Dose exposure and compliance, n (%)											
Adequate exposure	79 (85.9)	28 (84.8)	11 (84.6)	χ²= 0.04	0.978	85.80%	86.20%	86.00%	0.011	χ² = 0.01	0.995
Suboptimal exposure	13 (14.1)	5 (15.2)	2 (15.4)			14.20%	13.80%	14.00%			
Cumulative platinum dose (mg/m²), median (IQR)	395.0 (380.0-420.0)	401.0 (375.0-418.0)	396.0 (380.0-415.0)	H = 0.62	0.733	395.0 (380.0-415.0)	401.0 (378.0-418.0)	392.0 (375.0-410.0)	0.024	H = 0.54	0.768

WT, wild-type; IQR, interquartile range; Max SMD, maximum standardized mean difference among all pairwise comparisons; LDH, lactate dehydrogenase; INPC, International Neuroblastoma Pathology Classification; INRG, International Neuroblastoma Risk Group. The “Statistic” column displays the Kruskal–Wallis H statistic (H) for continuous variables and the chi-square value (χ²) derived from Pearson’s chi-square test (or Fisher’s exact test, as appropriate) for categorical variables. In the weighted cohort, the frequencies are presented as weighted percentages to reflect the adjusted pseudopopulation distribution. A Max SMD value of < 0.100 indicates optimal covariate balance across all three subgroups.

### 3.2 Primary endpoint: objective response rate assessed by BICR

**Figure 2:**
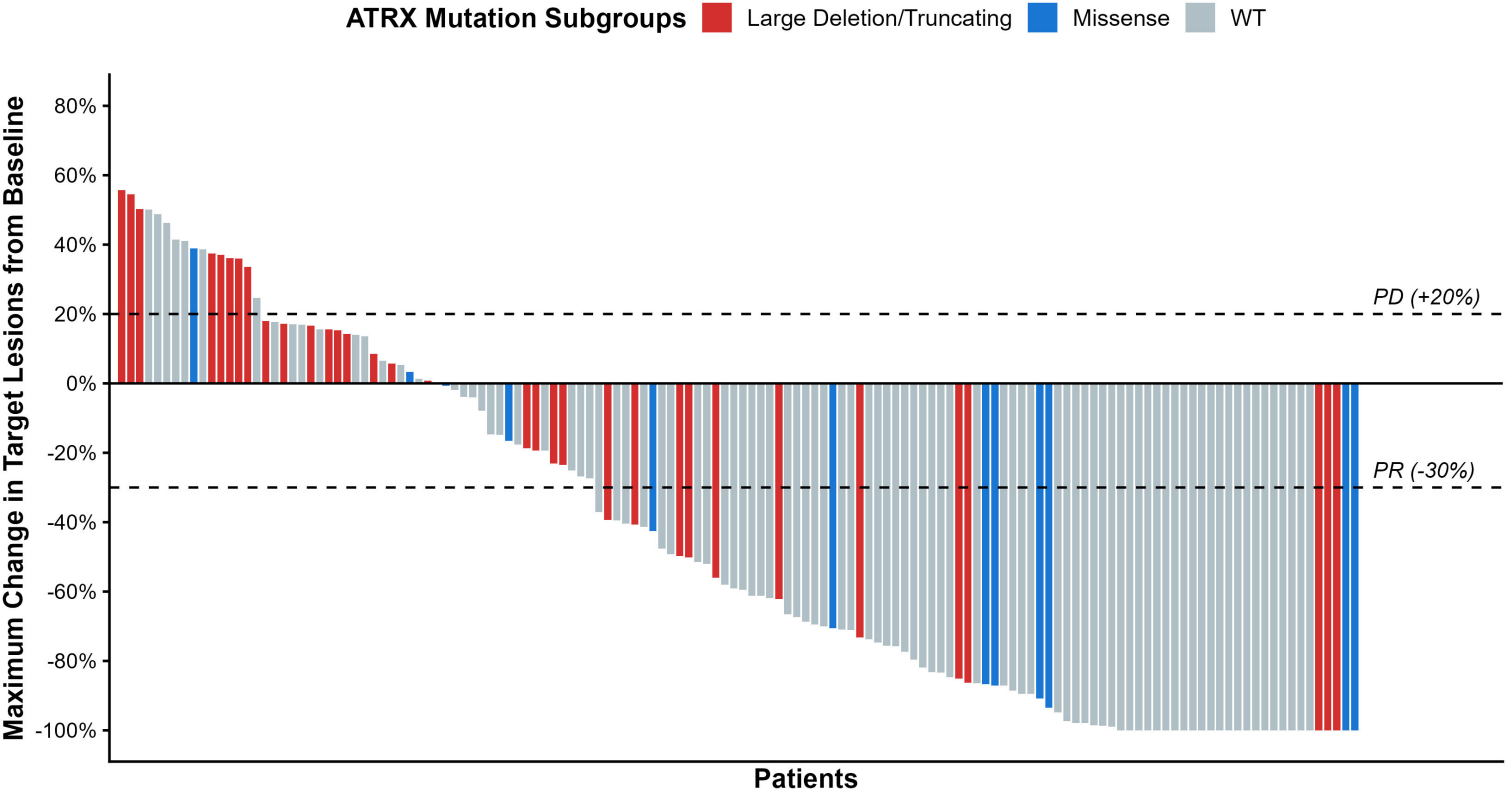
Waterfall plot of the best objective tumor response confirmed by blinded independent central review (BICR). The vertical axis represents the maximum percentage change in the sum of the longest diameters of the target lesions from baseline. The bars are color-coded according to the ATRX mutation subgroup (WT, large deletion/truncating, and missense). The dashed reference lines indicate the thresholds for partial response (-30%) and progressive disease (+20%) as defined by the revised INRC guidelines.

Efficacy outcomes, confirmed by the BICR according to the revised INRC guidelines, were formally compared across the molecular subgroups (**Figure 2**). In the IPTW-weighted pseudocohort, the large deletion/truncating group had a significantly lower ORR than the WT group (36.0% vs. 71.0%; P < 0.001) and a substantially higher primary induction-refractory rate (64.0% vs. 29.0%; P < 0.001). The missense mutation group demonstrated an intermediate ORR of 60.0%, which did not significantly differ from that of the WT cohort (P = 0.285) (**Table 2**). To rigorously eliminate the confounding quasicomplete separation induced by MYCN amplification, an independent analysis was conducted within the MYCN-NA subcohort (n = 95). Within this pure subset, the large deletion/truncating variant remained robustly associated with a diminished ORR (36.4%) relative to that of the WT group (70.0%). Subsequent multivariable logistic regression adjusted for 11q deletion status showed that large deletion/truncating ATRX alterations remained independently associated with a lower ORR in the MYCN-NA population (adjusted odds ratio = 0.28, 95% CI: 0.11–0.69; P = 0.006) (**Table 2**).

**Table 2:** Associations between ATRX mutation status and response to induction chemotherapy in the IPTW-weighted cohort and the MYCN-nonamplified subcohort.

Clinical Response (Revised INRC)	WT	Large Deletion/Truncating	Missense	Statistic	P value
IPTW-Weighted Cohort					
Complete Response (CR)	24.50%	9.50%	15.00%		
Partial Response (PR)	46.50%	26.50%	45.00%		
Minor Response (MR)	8.00%	15.00%	12.00%		
Stable Disease (SD)	13.00%	25.00%	18.00%		
Progressive Disease (PD)	8.00%	24.00%	10.00%		
Objective Response Rate (ORR)	71.00%	36.00%	60.00%	χ² = 15.82	< 0.001
WT vs. Large Deletion/Truncating				χ² = 14.65	< 0.001
WT vs. Missense				χ² = 1.14	0.285
Primary Induction-Refractory Rate*	29.00%	64.00%	40.00%	χ² = 14.95	< 0.001
MYCN-NA Subcohort (Unweighted, n=95)	(n = 50)	(n = 33)	(n = 12)		
Complete Response (CR), n (%)	12 (24.0)	3 (9.1)	2 (16.7)		
Partial Response (PR), n (%)	23 (46.0)	9 (27.3)	5 (41.7)		
Minor Response (MR), n (%)	4 (8.0)	5 (15.2)	1 (8.3)		
Stable Disease (SD), n (%)	7 (14.0)	8 (24.2)	3 (25.0)		
Progressive Disease (PD), n (%)	4 (8.0)	8 (24.2)	1 (8.3)		
Objective Response Rate (ORR)	35 (70.0)	12 (36.4)	7 (58.3)	χ² = 10.51	0.005
WT vs. Large Deletion/Truncating				χ² = 9.87	0.002
WT vs. Missense				χ² = 0.62	0.431

WT, wild-type; INRC, International Neuroblastoma Response Criteria; IPTW, inverse probability of treatment weighting; MYCN-NA, MYCN-nonamplified. In the IPTW-weighted cohort, the data are presented as weighted percentages. The “Statistic” column displays the chi-square value (χ²) derived from the weighted chi-square test (for the IPTW cohort) or Pearson’s chi-square/Fisher’s exact test (for the MYCN-NA subcohort). The objective response rate (ORR) encompasses CR and PR. *Primary induction-refractory disease comprised MR, SD, or PD at the end-of-induction assessment, together with documented disease progression necessitating premature discontinuation or modification of the planned induction regimen.

### 3.3 Secondary endpoints: event-free survival and cumulative incidence of progression

**Figure 3:**
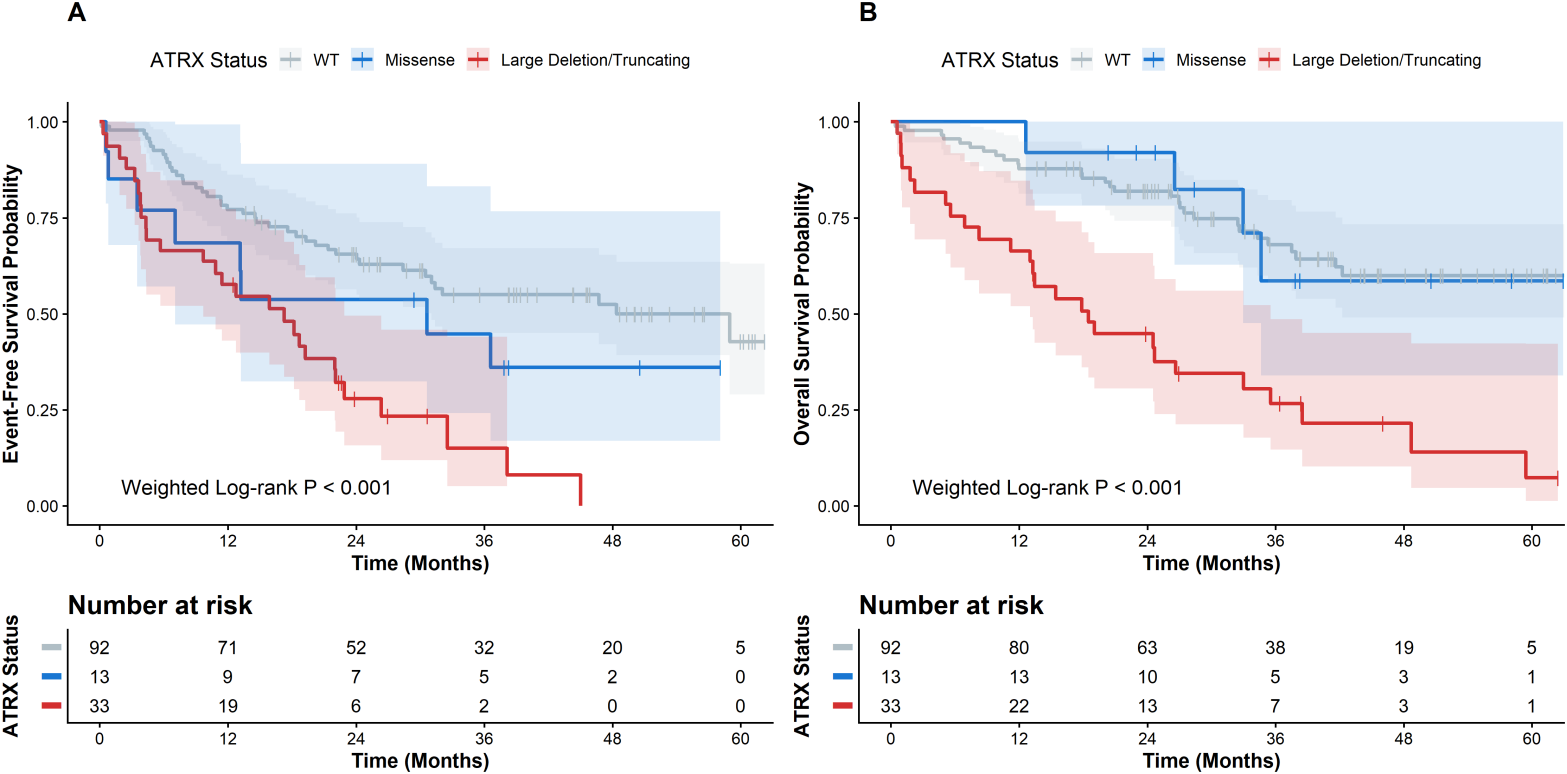
Kaplan–Meier curves for event-free survival (EFS) and overall survival (OS) stratified by ATRX mutation status in the IPTW-weighted cohort. **(A)** Event-free survival and **(B)** overall survival. The solid lines represent the survival estimates for the wild-type (WT), large deletion/truncating, and missense groups. Shaded areas denote the corresponding 95% confidence intervals. Tick marks indicate censored patients. P values were calculated using the weighted log-rank test.

**Figure 4:**
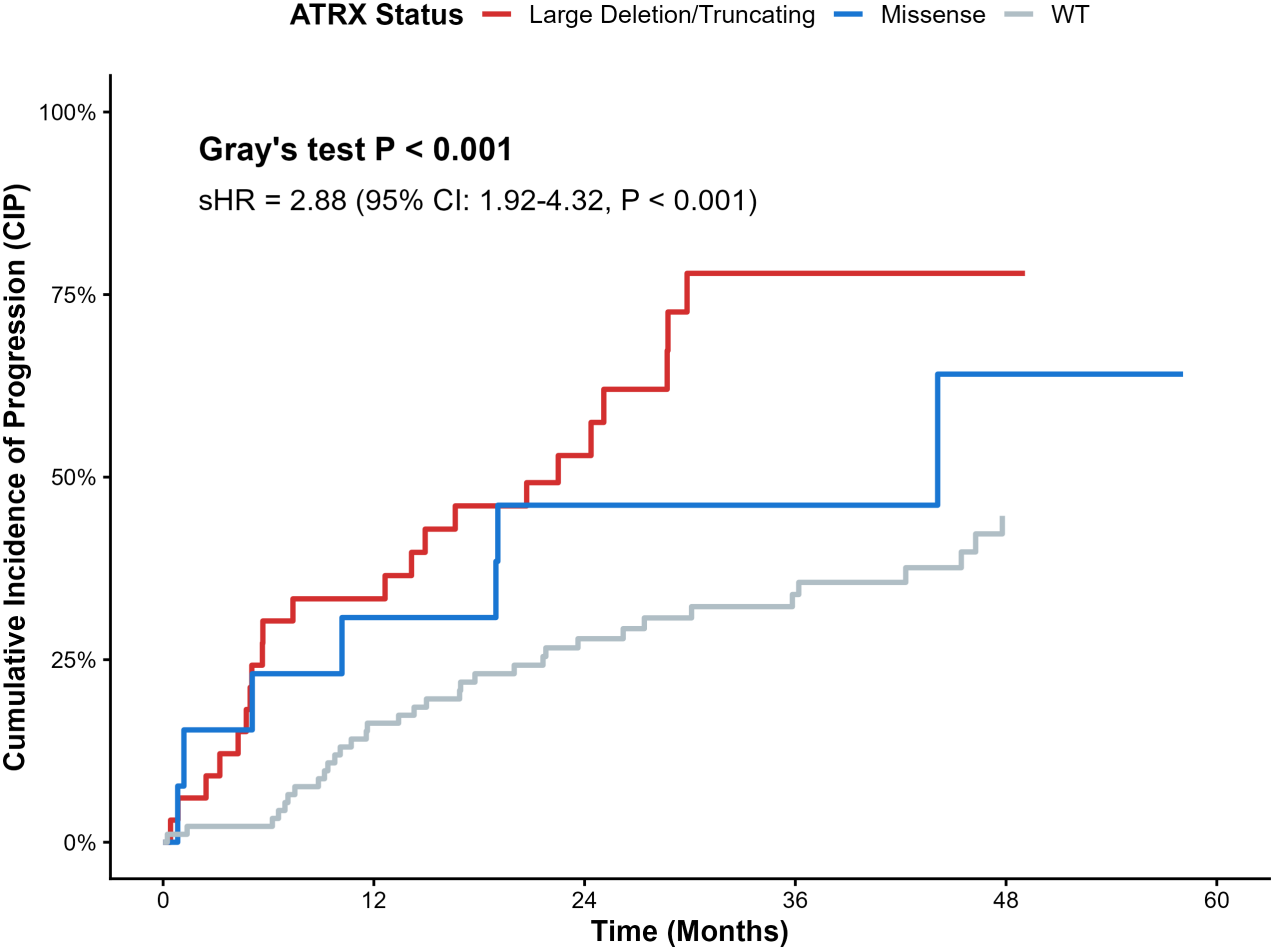
Cumulative incidence of progression (CIP) curves adjusted by the fine–gray competing risk model in the IPTW-weighted cohort. The curves illustrate the absolute probability of disease progression over time across the three molecular subgroups. Nontumor-specific mortality (e.g., lethal treatment-related toxicity) was strictly treated as a competing risk event. P values were derived from Gray’s test. The subdistribution hazard ratio (sHR) was calculated to quantify the relative risk of progression for the large deletion/truncating group vs. the WT group.

Survival outcomes were systematically evaluated within the IPTW-weighted pseudocohort. At a median follow-up of 42.5 months, compared with both the WT and missense cohorts, the large deletion/truncating group demonstrated significantly inferior survival trajectories. Specifically, the 3-year EFS rates were 18.5% for the large deletion/truncating group, 52.4% for the WT group, and 46.2% for the missense group (weighted log-rank P < 0.001). Consistent with the EFS findings, the 3-year OS rates were notably lower in patients harboring large deletions or truncating variants (28.4%) than in those in the WT (68.5%) and missense (61.0%) groups (weighted log-rank P < 0.001) (**Figure 3**). To rigorously account for the competing risk of non-tumor-specific mortality, particularly severe chemotherapy-induced toxicity prior to documented tumor progression, a Fine-Gray competing risk regression analysis was performed. This analysis revealed that the 3-year CIP was markedly greater in the large deletion/truncating group (72.6%) than in the WT group (35.2%; Gray’s test P < 0.001), with an estimated subdistribution hazard ratio (sHR) of 2.88 (95% CI: 1.92–4.32; P < 0.001) (**Figure 4**).

### 3.4 Subgroup and sensitivity analyses

**Figure 5:**
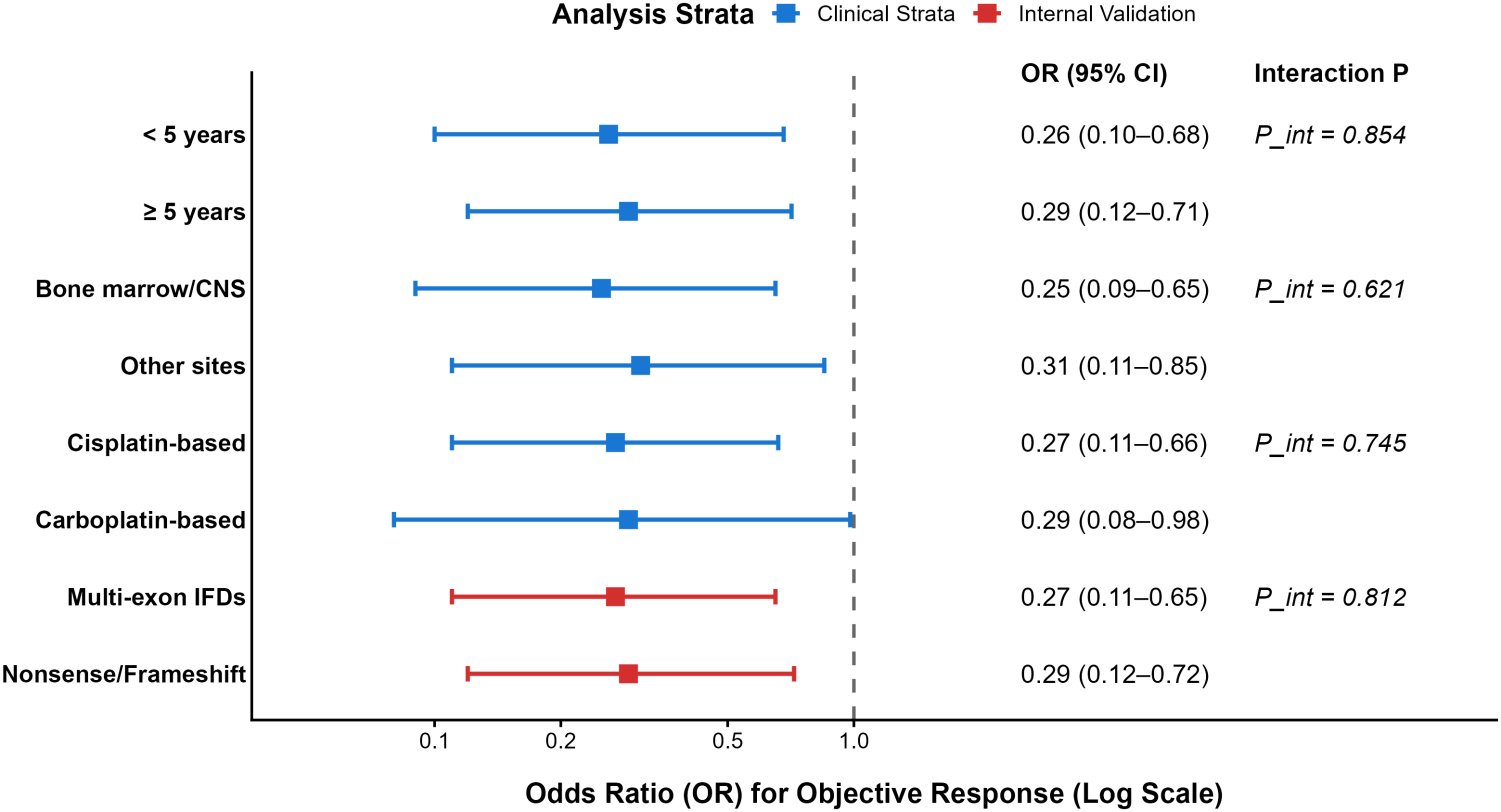
Forest plot of the association between large deletion/truncating ATRX mutations and the objective response rate to induction chemotherapy across predefined subgroups. The plot displays odds ratios (ORs) and 95% confidence intervals (CIs) for comparisons between the large deletion/truncating group and the WT group. In addition to clinical strata, including age, metastatic site, and the platinum agent included in the induction regimen, the plot includes an internal molecular comparison between multiexon IFDs and nonsense/frameshift variants. The vertical dashed line represents an OR of 1.0. P values for interactions indicate the consistency of the mutation effect across all evaluated subgroups.

Subgroup analyses demonstrated that the association between large ATRX deletion/truncating variants and an inferior ORR remained consistent across all predefined clinical strata (**Figure 5**). Specifically, a negative impact was observed regardless of age at diagnosis (< 5 years: OR 0.26; ≥ 5 years: OR 0.29), metastatic burden (bone marrow/CNS: OR 0.25; other sites: OR 0.31), or platinum agent included in the induction regimen (cisplatin-based: OR 0.27; carboplatin-based: OR 0.29), with all interaction P values > 0.05. Notably, an exploratory internal subgroup analysis within the large deletion/truncating cohort revealed that the deleterious effect on treatment response was equivalent between multiexon IFDs (OR 0.27, 95% CI: 0.11–0.65) and nonsense/frameshift variants (OR 0.29, 95% CI: 0.12–0.72), supporting the functional consolidation of these molecular subtypes (P for interaction = 0.812).

Two sensitivity analyses further confirmed the robustness of the primary findings (**Supplementary Table 1**). After weight trimming was applied to the IPTW model (excluding the 1st and 99th percentiles), the ORR remained significantly lower in the large deletion/truncating group than in the WT group (36.4% vs. 70.8%, P < 0.001). Similarly, in the “perfect compliance” cohort (n = 118), the ORR disparity persisted (37.5% in the large deletion/truncating group vs. 71.8% in the WT group, P < 0.001), indicating that the observed association was not explained by measured differences in chemotherapy delivery or extreme propensity weights.

### 3.5 Independent prognostic value of ATRX mutation subtypes

To determine the independent prognostic significance of the ATRX mutation subtypes, univariate and multivariable Cox proportional hazards regression analyses were performed using the original unweighted cohort (n = 138). In the multivariable model adjusted for MYCN amplification, 11q deletion, age at diagnosis, and chemotherapy dose exposure, large ATRX deletion/truncating variants were confirmed as independent risk factors for both inferior EFS (adjusted hazard ratio [aHR] = 3.42, 95% CI: 2.15–5.44, p < 0.001) and OS (aHR = 3.12, 95% CI: 1.88–5.18, p < 0.001) (**Table 3**). Notably, the interaction term between ATRX status and 11q deletion was not statistically significant (p = 0.428 for EFS; p = 0.512 for OS), indicating that the adverse prognostic impact of ATRX structural alterations is independent of 11q genomic status. Conversely, ATRX missense mutations were not significantly independently associated with survival outcomes (EFS: aHR = 1.24, p = 0.412; OS: aHR = 1.18, p = 0.584). The proportional hazards assumption was satisfied for all multivariable models on the basis of Schoenfeld residual testing.

**Table 3:** Univariate and multivariate Cox regression analyses of prognostic factors for EFS and OS in the original cohort (n = 138).

Variable	Univariate EFS		Multivariable EFS		Multivariable OS	
	HR (95% CI)	p value	aHR (95% CI)	p value	aHR (95% CI)	p value
ATRX Status						
Wild-type	Ref.	--	Ref.	--	Ref.	--
Large Deletion/Truncating	4.15 (2.85-6.04)	< 0.001	3.42 (2.15-5.44)	< 0.001	3.12 (1.88-5.18)	< 0.001
Missense	1.35 (0.72-2.54)	0.352	1.24 (0.65-2.36)	0.412	1.18 (0.58-2.42)	0.584
MYCN status						
Nonamplified	Ref.	--	Ref.	--	Ref.	--
Amplified	5.12 (3.48-7.52)	< 0.001	4.45 (2.95-6.72)	< 0.001	4.05 (2.55-6.42)	< 0.001
11q deletion						
No	Ref.	--	Ref.	--	Ref.	--
Yes	2.58 (1.75-3.81)	< 0.001	1.42 (0.92-2.18)	0.114	1.35 (0.85-2.14)	0.205
Age at diagnosis						
< 5 years	Ref.	--	Ref.	--	Ref.	--
≥ 5 years	2.84 (1.92-4.20)	< 0.001	1.55 (0.98-2.45)	0.062	1.48 (0.88-2.48)	0.142
Dose exposure						
Adequate	Ref.	--	Ref.	--	Ref.	--
Suboptimal	1.95 (1.25-3.04)	0.003	1.28 (0.82-2.02)	0.284	1.22 (0.75-1.98)	0.426

EFS, event-free survival; OS, overall survival; HR, hazard ratio; aHR, adjusted hazard ratio; CI, confidence interval; Ref., reference category. The multivariable model also included an interaction term between ATRX status and 11q deletion (interaction p = 0.428 for EFS, p = 0.512 for OS), which did not reach statistical significance. Chemotherapy dose exposure was categorized on the basis of the 80% dose and 4-week delay criteria.

## 4 Discussion

This multicenter retrospective cohort study delineated the prognostic and therapeutic implications of ATRX mutation subtypes in children and adolescents with high-risk neuroblastoma treated with platinum-containing multiagent induction chemotherapy. The principal finding was that the clinical associations of ATRX alterations varied according to mutation subtype: patients harboring large deletions or truncating mutations, including characteristic multiexon in-frame deletions, had substantially lower objective response rates, higher primary induction-refractory rates, and inferior event-free and overall survival compared with patients with ATRX wild-type tumors. In contrast, patients with ATRX missense mutations demonstrated intermediate outcomes that did not differ significantly from those of the wild-type cohort. These associations persisted after adjustment for age, MYCN amplification, 11q deletion status, and measured treatment exposure and were supported by sensitivity analyses restricted to patients with adequate treatment delivery. Because platinum compounds were administered together with other cytotoxic agents, these findings should be interpreted as evidence of resistance to the overall induction regimen rather than resistance specifically attributable to platinum agents.

Previous investigations have established that ATRX alterations define a distinct high-risk neuroblastoma subtype characterized by older age at diagnosis, mutual exclusivity with MYCN amplification, and an indolent yet inexorably progressive disease course associated with poor long-term survival. Kurihara and colleagues initially reported that ATRX-altered tumors without MYCN amplification detected beyond 18 months of age had poor prognosis and chemoresistance (7). More recently, a systematic review by Gonzales-Céspedes and colleagues revealed that ATRX and TERT aberrations constitute key biomarkers for accurate prognostic stratification in high-risk neuroblastoma (3). Lorenzi and colleagues further characterized the ATRX-mutant subgroup as presenting with chemotherapy-resistant, slowly progressive disease and demonstrated that ATRX alterations upregulate inflammatory pathways and mediate macrophage infiltration, revealing previously unrecognized immunogenic properties of these tumors (6). However, the literature has produced conflicting results regarding the precise magnitude of the adverse prognostic effect attributable to ATRX mutations. This ambiguity stems largely from the historical practice of aggregating all ATRX mutations into a single analytic category without consideration of their diverse structural and functional consequences. Our study addresses this inconsistency by performing fine-grained molecular subtyping on the basis of the predicted functional impact of each ATRX alteration. A recent dissertation by van Gerven systematically characterized the ATRX mutational landscape in neuroblastoma and reported that patients with ATRX missense mutations demonstrated slightly better overall survival than those with other mutation types did, a finding that aligns closely with our observation that missense mutations confer a neutral prognostic effect (9). By stratifying patients into large deletion/truncating, missense, and wild-type subgroups, our analysis reconciles the apparent prognostic heterogeneity reported in prior studies, highlighting that structural alterations involving extensive genomic loss constitute the predominant drivers of the aggressive clinical phenotype in this context.

The biological basis for the poor response to induction chemotherapy observed in the large deletion/truncating subgroup may reside in the unique molecular consequences of multiexon in-frame deletions that predominate in neuroblastoma. Unlike typical loss-of-function mutations that trigger nonsense-mediated mRNA decay and complete protein ablation, these characteristic multiexon deletions frequently escape nonsense-mediated decay and translate into internally deleted but partially functional truncated proteins. Recent genomic profiling studies have shown that 70% of ATRX aberrations in neuroblastoma are multiexon deletions, of which 75% are predicted to be in-frame and produce shortened protein products, a pattern that appears almost exclusive to neuroblastoma compared with other pediatric cancers (8, 9). Furthermore, these distinct aberrations produce divergent gene expression signatures; for instance, ATRX exon 2–10 multiexon deletion models exhibit reduced expression of genes involved in ribosome biogenesis and metabolism, confirming the existence of functionally distinct ATRX subtypes (9, 20). These aberrantly truncated proteins may exert dominant-negative effects that disrupt normal chromatin remodeling functions while retaining partial activity, thereby generating a unique cellular state characterized by defective DNA damage response signaling, altered replication fork dynamics, and aberrant telomere maintenance (21). ATRX plays a central role in depositing the histone variant H3.3 at repetitive genomic regions, including telomeres and pericentric heterochromatin, and its deficiency leads to telomere dysfunction, which activates the alternative lengthening of telomeres (ALT) pathway (17). Hartlieb and colleagues demonstrated that ALT-positive tumors display reduced ATRX/DAXX complex abundance and are molecularly distinct from other neuroblastoma subtypes (20), whereas Djos and colleagues corroborated a uniform correlation between ATRX aberrations and the ALT biomarker C-circles (10). Several agents used during induction, including platinum compounds, exert cytotoxic effects through DNA damage and replication stress. In ATRX-deficient cells, chronic replicative stress, ALT-mediated genomic instability, and a characteristically slow-cycling biological state may collectively reduce sensitivity to DNA-damaging multiagent chemotherapy. This altered cell cycle dynamics and the potential upregulation of damage tolerance pathways enable tumor cells to survive despite intensive induction therapy. Although ATRX deficiency may alter sensitivity to DNA-damaging agents, George and colleagues demonstrated that ATRX-mutant neuroblastoma cells harbor specific vulnerabilities in the DNA damage response network and exhibit selective sensitivity to PARP and ATM inhibition (22).while A recent study by Lorenzi and colleagues further revealed that ATRX in-frame deletions and loss-of-function mutations elicit differential chromatin accessibility responses, underscoring the functional divergence between mutation subtypes and their potential impact on therapeutic response (23).

A critical methodological challenge in assessing the independent prognostic value of ATRX alterations lies in disentangling their effects from those of tightly linked genomic covariates. Our data confirmed that ATRX structural alterations cooccur at a high frequency with 11q deletion, with 72.7% of the large deletion/truncating group harboring concurrent 11q loss compared with 23.9% of wild-type patients. This enrichment pattern has been well documented, as 11q deletions are known to cooccur more frequently with ATRX multiexon deletions than with point mutations or wild-type tumors (8, 9). To rigorously address this collinearity, our multivariable Cox regression model included an interaction term between ATRX status and 11q deletion. The absence of a statistically significant interaction indicated that the adverse prognostic impact of ATRX structural alterations operates independently of 11q genomic status, establishing that ATRX disruption is an autonomous driver of poor outcome rather than a mere passenger event in 11q-deleted tumors. Furthermore, the near-complete mutual exclusivity between ATRX mutations and MYCN amplification observed in our cohort mirrors the incompatibility previously characterized in preclinical models. Zeineldin and colleagues demonstrated that elevated MYCN expression and ATRX mutations are incompatible in neuroblastoma, leading to metabolic reprogramming and lethal replicative stress (24). By restricting the analysis to the MYCN-nonamplified subcohort, we reduced confounding related to MYCN amplification and found that LDT ATRX alterations remained independently associated with a lower ORR to induction chemotherapy.

The clinical implications of these findings are potentially important. Contemporary induction regimens for high-risk neuroblastoma use platinum compounds as components of multiagent chemotherapy. In our cohort, patients harboring large ATRX deletions or truncating mutations responded poorly to these conventional induction regimens, with an objective response rate of 36% and a primary induction-refractory rate of 64%. These findings support the incorporation of detailed ATRX mutation subtyping into baseline molecular profiling to facilitate the early identification of patients at high risk of inadequate induction response. However, because all patients received combination chemotherapy, the present study does not establish that omission or substitution of the platinum component would improve outcomes. Preclinical evidence suggests that ATRX-mutant neuroblastoma may have therapeutic vulnerabilities involving PARP, ATM, ATR, or WEE1-related pathways (21, 22, 25). Future prospective studies should therefore evaluate molecularly adapted or early response-guided induction strategies and determine whether modifying individual cytotoxic components or incorporating targeted agents improves outcomes in patients with ATRX structural alterations.

Several limitations of this study warrant careful consideration. First, despite the implementation of multiple imputation, inverse probability of treatment weighting, and rigorous sensitivity analyses, the retrospective design inherently precludes the complete elimination of unmeasured residual confounding. Second, although our stratified random sampling strategy successfully generated a balanced analytic cohort, the absolute number of patients in the missense mutation group remained modest, raising the possibility of a type II error with respect to the neutral prognostic effect attributed to this subgroup. Validation in larger international collaborative cohorts is essential to confirm the generalizability of these findings. Third, the absence of paired *in vitro* or *in vivo* functional validation experiments within this study precludes the definitive mechanistic attribution of the observed resistance phenotype to specific molecular pathways. Fourth, although central reannotation of original raw sequencing data was not performed, the use of standardized ACMG criteria for variant interpretation and BICR for imaging endpoints significantly mitigated this concern. Fifth, the long-term follow-up period, while adequate for assessing induction response and early survival outcomes, may not fully capture the chronic but progressive disease trajectory characteristic of ATRX-mutant neuroblastoma, and extended surveillance is warranted. Sixth, although we adjusted for the cumulative platinum dose, treatment delays, and categorical treatment exposure, detailed agent-specific relative dose intensity was not uniformly available. Moreover, because platinum compounds were administered exclusively as components of multiagent induction regimens, the present study could not separate their contribution from that of the accompanying cytotoxic agents. Consequently, the observed phenotype should be interpreted as resistance to conventional induction chemotherapy rather than as platinum-specific resistance. Finally, the study population was derived from institutions with an established molecular profiling infrastructure, which may limit its applicability to settings with more limited genomic testing capabilities.

## 5 Conclusion

This study showed that large ATRX deletions and truncating mutations, including multiexon in-frame deletions, identify an ultrahigh-risk molecular subtype of neuroblastoma characterized by poor response to conventional multiagent induction chemotherapy, accelerated disease progression, and markedly inferior survival outcomes. These associations were independent of age, MYCN amplification, 11q deletion status, and measured treatment exposure. Detailed ATRX mutation subtyping may therefore improve early risk stratification and facilitate the prospective evaluation of molecularly adapted or response-guided induction strategies. Because platinum compounds were administered as components of combination regimens, further treatment-specific and functional studies are required to determine whether the resistance phenotype is selectively attributable to platinum agents.

## Data Availability

The raw data supporting the conclusions of this article will be made available by the authors, without undue reservation.
