# Supplementary material for: ATRX large deletions and truncating mutations are associated with primary induction chemotherapy resistance in high-risk neuroblastoma: a multicenter propensity-weighted cohort study
Source: Front Oncol. 2026 Jul 22;16:1857869. doi: 10.3389/fonc.2026.1857869 (PMC13437291; doi:10.3389/fonc.2026.1857869)
Supplement: Supplementary file 1 [file Table1.docx]

**Supplementary Table 1.** Sensitivity analysis evaluating the robustness of ORR outcomes across different statistical and clinical constraints

| **Analysis Strategy** | **WT Group ORR, % (n/N)** | **Large Deletion/ Truncating ORR, % (n/N)** | **Missense Group ORR, % (n/N)** | **Statistic (Weighted X2)** | **P-value** |
| --- | --- | --- | --- | --- | --- |
| Primary Analysis (Full IPTW Cohort) | 71.0% (65.3/92.0) | 36.0% (11.9/33.0) | 60.0% (7.8/13.0) | 15.82 | < 0.001 |
| Sensitivity Analysis 1: Weight Trimming | 70.8% (63.7/90.0) | 36.4% (11.3/31.0) | 59.5% (7.1/12.0) | 15.24 | < 0.001 |
| Sensitivity Analysis 2: Perfect Compliance | 71.8% (56.7/79.0) | 37.5% (10.5/28.0) | 63.6% (7.0/11.0) | 12.65 | < 0.001 |

Notes: WT, wild-type; IPTW, inverse probability of treatment weighting; ORR, objective response rate. In the primary and weight-trimming analyses, n/N represents weighted frequencies and total effective sample sizes. Perfect compliance cohort is defined as patients receiving ≥ 80% of the planned dose with ≤ 4 weeks delay. P-values represent the global comparison across the three groups.
